# Family Support to Women During Pregnancy and Its Impact on Maternal and Fetal Outcomes

**DOI:** 10.7759/cureus.62002

**Published:** 2024-06-09

**Authors:** Ujwala R Mane, Jyoti A Salunkhe, Satish Kakade

**Affiliations:** 1 Community Health Nursing, Krishna Institute of Nursing Sciences, Krishna Vishwa Vidyapeeth (Deemed to be University), Karad, IND; 2 Obstetrics and Gynecology, Krishna Institute of Nursing Sciences, Krishna Vishwa Vidyapeeth (Deemed to be University), Karad, IND; 3 Community Medicine, Krishna Institute of Nursing Sciences, Krishna Vishwa Vidyapeeth (Deemed to be University), Karad, IND

**Keywords:** fetomaternal outcome, women of reproductive age group, first trimester pregnancy, family relationship, fetal outcome, patient-family centered care

## Abstract

Background: Family support is one of the determinants of lifestyle habits and relevant health behavior for pregnancy outcomes. In India, the joint family system is still practiced. Due to education, urbanization, and industrialization, the family institution continues to play a central role in people's lives. Pregnancy is a crucial period in women’s lives. Good care during pregnancy is important for the health of the mother and the newborn baby. During this period, hormonal changes are complex and involve multiple hormones working together to support the developing fetus and prepare the mother's body for labor, delivery, and breastfeeding. To avoid maternal and fetal complications, she needs support from her family throughout pregnancy and the postnatal period.

Aim and objectives: This study aims to evaluate the influence of the level and quality of family support during pregnancy on maternal and fetal outcomes and to identify any association between the sociodemographic variables and the impact of the level and quality of family support during the first trimester.

Material and methods: This study used a quantitative approach with a survey research design. Data were collected from four Primary Health Centers at Karad, Maharashtra, India, i.e., Rethare, Vadgaon, Kale, and Supane. A consecutive sampling technique was used to select the 344 subjects from the Rethare, Vadgaon, Kale, and Supane areas of Karad Taluka. Data were collected before the completion of the first three months of pregnancy, then during the second trimester and after delivery. Upon evaluation, the tool was validated by experts representing a range of specialties, including community health nursing, mental health nursing, obstetric gynecology, and pediatric care. A pilot study was conducted on 30 samples. The data collected were analyzed by using descriptive and inferential statistics.

Result: The findings of the study show a significant association between the psychosocial support received in the first trimester and the total gestational weeks completed at the time of delivery (p < 0.05). The study suggests the need for psychosocial support during the first trimester for better maternal and fetal outcomes.

Conclusion: Psychosocial family support is needed by pregnant women during the first trimester to achieve maternal and fetal outcomes.

## Introduction

Understanding whether low support is responsible for the increased risk of preterm birth could help health professionals identify women early in pregnancy and connect them with appropriate support [1]. Children of pregnant women tend to have low birth weight (LBW), fail to grow at a normal rate, and have higher rates of obtaining disease, potentially leading to early death due to lack of family support [2]. Throughout pregnancy, women need support from the family to gain better maternal and fetal outcomes [3]. Emotional distress in women during pregnancy has been shown to increase the risk of adverse outcomes for women and newborns [4]. Maternal mortality is unacceptably high. About 2,95,000 women died during and after pregnancy and childbirth in 2017. Most of these deaths (94%) occurred in low-resource settings, and most could have been prevented [5]. Family support, including the husband's role as the head of the family, is crucial during pregnancy. Pregnancy, the longest nine-month period of their lives, is a period characterized by heightened emotions, and caring for a newborn can be exhausting. During this time, individuals need to have the support of close relatives who can assist with caring for both the mother and the baby. Helping these relationships to meet health needs is important for the woman and the newborn baby [6].

## Materials and methods

The present study was conducted at four Primary Health Centres (PHCs) in the rural area of Karad Taluka, Maharashtra, India, from 2021 to 2023. There are 11 PHCs in Karad Taluka; initially, out of 11 PHCs, a random selection of four PHCs was done. The desired 344 samples were collected from 40 selected villages. The participants for the current study included first-time and multiple-time pregnant women aged 18-30 years who were residents registered and gave birth at the selected PHCs of Karad Taluka or at Krishna Hospital Karad.

**Figure 1:**
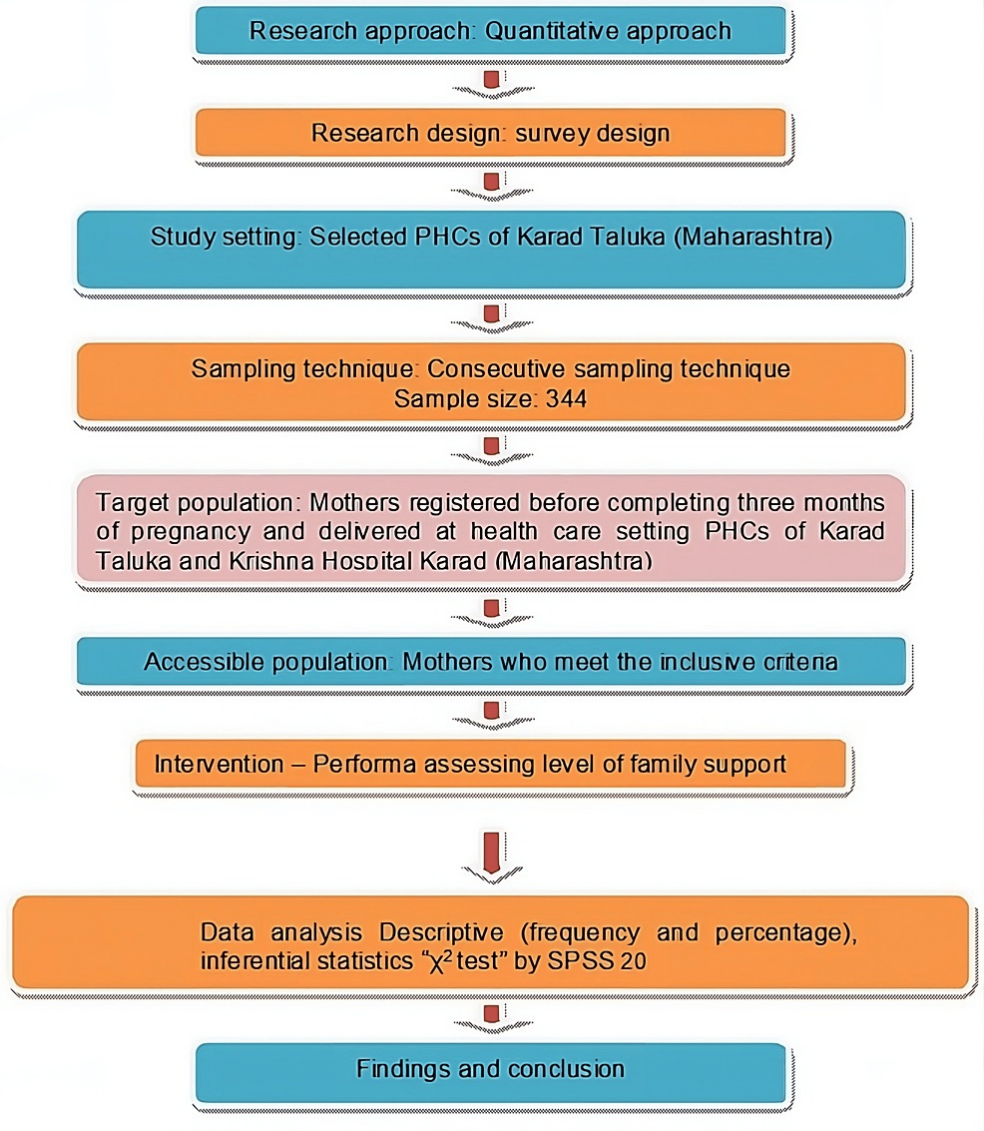
Schematic of the research process

Ethical permission for the study was obtained through Krishna Vishwa Vidyapeeth (Deemed to be University), registration number KIMSDU/PhD/Adm./13/2020. Women were enrolled using a consecutive sampling technique. This method involved enrolling all eligible women from conception until three months of pregnancy, provided they met the study's inclusion and exclusion criteria. Enrollment was continuous as these women visited the selected PHCs in Karad Taluka. During the initial visit, participants underwent screening according to the inclusion criteria, and informed consent was obtained before enrolling pregnant women in the study. Data were collected during three subsequent visits: the first visit within the first three months after conception, the second during the second trimester, and the final visit after delivery. The independent variable was the level of family support, and the dependent variable was the maternal and fetal outcomes. In the first trimester, 103 (29.9%) women received strong support, 175 (50.9%) received moderate support, and 66 (19.2%) received poor family support, encompassing physical, emotional, and psychosocial aspects. Figure 1 shows the schematic of the research design.

The sampling technique involves selecting representative units of the target population. It is the process of choosing a portion of the population. The study utilized a consecutive sampling technique. The sample size of 344 pregnant women was calculated based on the findings of the study conducted by Abdollahpour et al. [7]. The proportion of pregnancy complications observed in women [7] with poor support from family was 81.8%, while the proportion of pregnancy complications in women with moderate or good support from families was 45.2%. The pregnant woman who needed to be enrolled in the current study was determined as follows:

$n =\frac{(p1q1+p2q2)(Z1-\alpha /2+Z1-\beta ){^{2}}}{(p1-p2)^{2}}$

where p1 is the proportion of women having pregnancy complications with poor/moderate family support; q1 = 100-p1; p2 is the proportion of women having pregnancy complications with good family support; q2 = 100-p2; Z1-œ/2 = level of significance (5%), i.e., = 1.96; Z1-ß = power of the study (95%), i.e., 1.64; i.e. = 1.96Z1-ß = power of the study (95%), i.e., =1.64. Thus,

$n=\frac{(81.8 &times; 18.2)+(45.2 &times; 54.8) &times; 13}{(81.8 - 45.2)^{2}}$

n = 39

Approximately 10% of women might not complete the follow-up periods from conception to the postpartum period in the study. A minimum of 43 (i.e., 39 + 3) women with varying levels of family support (poor, moderate, and good) were enrolled from four randomly selected PHCs under Karad Taluka. There are 11 PHCs in Karad Taluka, out of which four PHCs were chosen randomly for the study. The sample size is n = 344.

Criteria for the selection of the sample

The inclusion criteria concerned pregnant women aged 18 to 30, primigravida and multigravida women, who visited selected PHCs under Karad Taluka, registered within three months of pregnancy and delivered under allotted PHCs, health care settings, or Krishna Hospital, Karad.

Exclusion criteria included pregnant women who suffered severe disorders or disability during the first trimester, had a history of drug or alcohol abuse, were diagnosed with mental illnesses, or were not interested.

Data collection instrument: The pregnancy outcome was categorized based on the type of delivery: preterm (<37 weeks of pregnancy), term (37-42 weeks of pregnancy), and post-term delivery (>42 weeks of pregnancy). Mode of delivery options included spontaneous vaginal delivery, assisted vaginal delivery, lower segment cesarean section, or forceps/vacuum delivery. Fetal outcome encompasses the baby's birth weight, with an LBW baby defined as having a birth weight of less than 2.5 kg and a normal baby having a birth weight of 2.5 kg or greater. The baby's birth status includes whether the baby was born normally or had any complications. The length of the baby and family support received for baby care were calculated. The quality and level of family support, including physical, emotional, and psychosocial support, should be categorized as poor, moderate, or good during each trimester and the postnatal period.

## Results

Table 1 shows that 50 women (14.5%) received good physical support during the first trimester, 196 (57%) received moderate physical support, and 98 (28.5%) received poor physical support during the first trimester. During the first trimester, 65 (18.9%) pregnant women received good emotional support, 169 (49.1%) received moderate support, and 110 (32%) received poor support. According to the psychosocial support survey, 56 individuals (16.3%) reported receiving good psychosocial support, 181 (52.6%) reported receiving moderate support, and 107 (31.1%) reported receiving poor support. In total, in the first trimester, 103 (29.9%) received good family support, 175 (50.9%) received moderate support, and 66 (19.2%) received poor support.

**Table 1:** Distribution of pregnant women according to the level of family support during the first trimester (N = 344) N: sample size; n: number of participants included

Level of support	Score	Frequency (n)	Percentage (%)
Physical support			
Poor	≤8	98	28.5
Moderate	9-16	196	57
Good	17-20	50	14.5
Emotional support			
Poor	≤6	110	32
Moderate	7-12	169	49.1
Good	13-16	65	18.9
Psychosocial support			
Poor	≤6	107	31.1
Moderate	7-12	181	52.6
Good	13-16	56	16.3
Total support			
Poor	≤17	66	19.2
Moderate	8-34	175	50.9
Good	35-52	103	29.9

Table 2 shows the association between maternal outcomes and physical support during the first trimester. The results indicate no significant association between physical support during the first trimester and the following variables: gestational weeks completed at the time of delivery, presence of associated maternal complications/diseases during pregnancy, presence of close relatives before delivery, relationship with the attendee, presence of maternal complications during delivery, specific maternal complications, type of delivery, received family support during delivery, relationship with the attendee, and family support received after delivery (p > 0.05).

**Table 2:** Association between maternal outcome and the level of physical support during the first trimester (N = 344) P value was calculated by the chi-square method N: sample size; n: number of participants included; PPH: postpartum hemorrhage

Maternal outcome	Poor support, n (%)	Moderate support, n (%)	Good support, n (%)	Total, n (%)	Χ^2 ^value	P value
Total gestational weeks completed at the time of delivery						
≤36 weeks	33 (37.08)	42 (47.19)	14 (15.73)	89 (25.87)	6.31	0.177
37-40 weeks	58 (28)	141 (52)	31 (20)	230 (66.86)		
>40 weeks	7 (28)	13 (52)	5 (20)	25 (7.27)		
Presence of associated maternal complications/diseases during pregnancy						
Yes	30 (26.32)	64 (56.14)	20 (17.54)	114 (33.14)	1.366	0.505
No	68 (29.57)	132 (57.39)	30 (13.04)	230 (66.86)		
If yes, specify maternal complications						
PPH	1 (16.67)	4 (66.67)	1 (16.67)	6 (1.74)	4.45	0.814
Eclampsia	7 (18.42)	24 (63.16)	7 (18.42)	38 (11.05)		
Fever	1 (50)	1 (50)	0 (0)	2 (0.58)		
Other complications	21 (30.88)	35 (51.47)	12 (17.65)	68 (19.77)		
No complications	68 (29.57)	132 (57.39)	30 (13.04)	230 (66.86)		
Presence of close relatives before delivery						
Yes	73 (29.55)	138 (55.87)	36 (14.57)	247 (71.8)	0.539	0.764
No	25 (25.77)	58 (59.79)	14 (14.43)	97 (28.20)		
If yes, relationship with the attendee						
Mother	49 (35)	69 (49.29)	22 (15.71)	140 (40.7)	9.42	0.151
Husband	18 (22.5)	49 (61.25)	13 (16.25)	80 (23.26)		
Other relatives	6 (22.22)	20 (74.07)	1 (3.70)	27 (7.85)		
Mother-in-law	25 (25.77)	58 (59.79)	14 (14.43)	97 (28.20)		
Presence of associated maternal complications during delivery						
Yes	24 (26.67)	51 (56.67)	15 (16.67)	90 (26.16)	0.525	0.769
No	74 (29.13)	145 (57.09)	35 (13.78)	254 (73.84)		
If yes, specify maternal complications during delivery						
PPH	2 (33.33)	3 (50)	1 (16.67)	6 (1.74)	5.924	0.656
Eclampsia	2 (50)	2 (50)	0 (0)	4 (1.16)		
Infection	0 (0)	8 (80)	2 (20%)	10 (2.91)		
Others	20 (28.57)	38 (54.29)	12 (17.14)	70 (20.35)		
No complications	74 (29.13)	145 (57.09)	35 (13.78)	254 (73.84)		
Type of delivery						
Normal vaginal delivery	45 (25.57)	109 (61.93)	22 (12.50)	176 (51.16)	10.928	0.091
Episiotomy	26 (28.26)	46 (50)	20 (21.74)	92 (26.74)		
Cesarean mode of delivery	27 (36.99)	38 (52.05)	8 (10.96)	73 (21.22)		
Instrumental delivery	0 (0)	3 (0.87)	0 (0%	3 (0.87)		
Received family support during delivery						
Yes	89 (32.13)	144 (51.99)	44 (15.88)	277 (80.52)	0.724	0.696
No	18 (26.87)	37 (55.22)	12 (17.91)	67 (19.48)		
If yes, relationship with the attendee						
Mother	58 (35.37)	83 (50.61)	23 (14.02)	164 (47.67)	4.582	0.801
Husband	18 (26.09)	38 (55.07)	13 (18.84)	69 (20.06)		
Other relatives	12 (30)	20 (50)	8 (20)	40 (11.63)		
Mother-in-law	1 (25)	3 (75)	0 (0)	4 (1.16)		
No relatives	18 (26.87)	37 (55.22)	12 (17.91)	67 (19.48)		
Family support received after delivery						
Yes	83 (29.96)	157 (56.68)	37 (13.36)	277 (80.52)	2.466	0.291
No	15 (22.39)	39 (58.21)	13 (19.40)	67 (19.48)		
If yes, relationship with the attendee						
Mother	52 (31.71)	89 (54.27)	23 (14.02)	164 (47.67)	9.512	0.301
Husband	17 (24.64)	41 (59.42)	11 (15.94)	69 (20.06)		
Other relatives	11 (27.5)	26 (65)	3 (7.5)	40 (11.63)		
Mother-in-law	3 (75)	1 (25)	0 (0)	4 (1.16)		
No relatives	15 (22.39)	39 (58.21)	13 (19.40)	67 (19.48)		

Table 3 shows that there was no significant association found between gestational weeks completed at the time of delivery, presence of associated maternal complications/diseases during pregnancy, presence of close relatives before delivery, relationship with the attendee, presence of maternal complications during delivery, specific maternal complication, type of delivery, received family support during delivery, relationship with the attendee, family support received after delivery, relationship with the attendee, and emotional support during the first trimester.

**Table 3:** Association between maternal outcome and the level of emotional support during first trimester (N = 344) P value was calculated by the chi-square method N: sample size; n: number of participants included; PPH: postpartum hemorrhage

Maternal outcome	Poor/no support, n (%)	Moderate support, n (%)	Good support, n (%)	Total, n (%)	χ^2^ value	P value
Total gestational weeks completed at the time of delivery						
Below 36 weeks of gestation	29 (32.58)	39 (43.82)	21 (23.59)	89 (25.87)	3.609	0.461
37-40 weeks of gestation	72 (31.30)	120 (52.17)	38 (16.52)	230 (66.86)		
Above 40 weeks of gestation	9 (36)	10 (40)	6 (24)	25 (7.26)		
Presence of associated maternal complications/diseases during pregnancy						
Yes	32 (28.07)	60 (52.63)	22 (19.29)	114 (33.13)	1.254	0.534
No	78 (33.91)	109 (47.39)	43 (18.69)	230 (66.86)		
If yes, specify maternal complications						
PPH	1 (16.66)	4 (66.66)	1 (16.66)	6 (1.74)	4.361	0.823
Echlamsia	12 (31.57)	21 (55.26)	5 (13.15)	38 (11.04)		
Fever	1 (50)	1 (50)	0	2 (0.58)		
Other complications	19 (27.94)	32 (47.05)	17 (25)	68 (19.76)		
No complications	1 (16.66)	4 (66.66)	1 (16.66)	6 (1.74)		
Presence of close relatives before delivery						
Yes	76 (30.76)	125 (50.60)	46 (18.62)	247 (71.80)	0.824	0.662
No	34 (35.05)	44 (45.36)	19 (19.58)	97 (28.19)		
If yes, the relationship with the attendee						
Mother	47 (33.57)	68 (48.57)	25 (17.85)	140 (40.69)	6.157	0.406
Husband	18 (22.5)	47 (58.75)	15 (18.75)	80 (23.25)		
Other relatives	11 (40.74)	10 (37.03)	6 (22.22)	27 (7.84)		
Mother-in-law	34 (35.05)	44 (45.36)	19 (19.58)	97 (28.19)		
Presence of associated maternal complications during delivery						
Yes	27 (30)	47 (52.22)	16 (17.77)	90 (26.16)	0.467	0.792
No	83 (32.67)	122 (48.03)	49 (19.29)	254 (73.83)		
If yes, specify maternal complications during delivery						
PPH	2 (33.33)	2 (33.33)	2 (33.33)	6 (1.74)	7.297	0.505
Echlamsia	2 (66.66)	2 (66.66)	0 (00.00)	4 (1.16)		
Infection	1 (10)	5 (50)	4 (40)	10 (2.90)		
Others	22 (31.42)	38 (54.28)	10 (14.28)	70 (20.34)		
No complications	83 (32.67)	122 (48.03)	49 (19.29)	254 (73.83)		
Type of delivery						
Normal vaginal delivery	55 (31.25)	87 (49.43)	34 (19.31)	176 (51.16)	5.522	0.479
Episiotomy	29 (31.52)	42 (45.65)	21 (22.82)	92 (26.74)		
Cesarean mode of delivery	26 (35.61)	37 (50.68)	10 (13.69)	73 (21.22)		
Instrumental delivery	0 (0.00)	3 (100)	0 (0.00)	3 (0.87)		
Received family support during delivery						
Yes	89 (31.12)	141 (41.15)	47 (16.96)	277 (80.52)	3.72	0.156
No	21 (31.34)	28 (41.79)	18 (26.86)	67 (19.47)		
If yes, the relationship with the attendee						
Mother	54 (32.92)	84 (51.21)	26 (15.85)	164 (47.67)	8.153	0.419
Husband	22 (31.88)	31 (44.92)	16 (23.18)	69 (20.05)		
Other relatives	11 (27.25)	24 (60)	5 (12.5)	40 (11.62)		
Mother-in-law	2 (50)	2 (50)	0 (0.00)	4 (1.162)		
No relatives	21 (31.34)	28 (41.81)	18 (26.86)	67 (19.47)		
Family support received after delivery						
Yes	75 (29.18)	135 (52.52)	47 (18.28)	257 (74.70)	5.072	0.079
No	35 (40.22)	34 (39.08)	18 (20.68)	87 (25.29)		
If yes, the relationship with the attendee						
Mother	50 (29.76)	89 (52.97)	29 (17.261)	168 (48.83)	6.746	0.564
Husband	11 (26.19)	22 (52.38)	9 (21.42)	42 (12.20)		
Mother-in-law	12 (32.43)	19 (51.35)	6 (16.21)	37 (10.75)		
Other relatives	2 (20)	5 (50)	3 (30)	10 (2.90)		
No relatives	35 (40.22)	34 (39.08)	18 (20.68)	87 (25.290)		

Table 4 shows that there was a significant association found between psychosocial support received in the first trimester and total gestational weeks completed at the time of delivery (p < 0.05). No significant association was found between the presence of associated maternal complications/diseases during pregnancy, presence of close relatives before delivery, relationship with the attendee, presence of maternal complications during delivery, specific maternal complication, type of delivery, received family support during delivery, relationship with the attendee, and family support received after delivery with psychosocial support at the first trimester during pregnancy (p > 0.05).

**Table 4:** Association between maternal outcome and the level of psychosocial support during the first trimester (N = 344) P value was calculated by the chi-square method N: sample size; n: number of participants included; PPH: postpartum hemorrhage

Maternal outcome	Poor/no support, n (%)	Moderate support, n (%)	Good support, n (%)	Total, n (%)	Χ^2^ value	P value
Total gestational weeks completed at the time of delivery						
Below 36 weeks of gestation	28 (31.46)	39 (43.82)	22 (24.72)	89 (25.87)	11.768	0.019
37-40 weeks of gestation	67 (29.13)	133 (57.82)	30 (13.04)	230 (66.86)		
Above 40 weeks of gestation	12 (48)	9 (36)	4 (16)	25 (7.27)		
Presence of associated maternal complications/diseases during pregnancy						
Yes	29 (25.44)	64 (56.14)	21 (18.42)	114 (33.14)	2.643	0.267
No	78 (33.91)	117 (50.87)	35 (15.22)	230 (66.86)		
If yes, specify maternal complications						
PPH	1 (16.67)	2 (33.33)	3 (50)	6 (1.74)	11.328	0.184
Echlamsia	10 (26.31)	24 (63.16)	4 (10.52)	38 (11.04)		
Fever	0 (0)	1 (50)	1 (50)	2 (0.58)		
Other complications	17 (25)	38 (55.88)	13 (19.12)	68 (19.77)		
No complications	79 (34.35)	116 (50.43)	35 (15.22)	230 (66.86)		
Presence of close relatives before delivery						
Yes	78 (31.58)	128 (51.82)	41 (16.60)	247 (71.80)	0.224	0.894
No	29 (29.90)	53 (54.64)	15 (15.46)	97 (28.20)		
If yes, the relationship with the attendee						
Mother	42 (30)	76 (54.28)	22 (15.71)	140 (40.70)	2.958	0.814
Husband	28 (35)	36 (45)	16 (20)	80 (23.25)		
Other relatives	8 (29.63)	16 (59.26)	3 (11.11)	27 (7.85)		
No relatives	29 (29.90)	53 (54.64)	15 (15.46)	97 (28.20)		
Presence of associated maternal complications during delivery						
Yes	25 (27.78)	53 (58.89)	12 (13.33)	90 (26.16)	1.995	0.369
No	82 (32.28)	128 (50.39)	44 ()17.32	254 (73.84)		
If yes, specify maternal complications during delivery						
PPH	1 (16.67)	4 (66.67)	1 (16.67)	6 (1.74)	6.126	0.633
Echlamsia	2 (50)	2 (50)	0 (0)	4 (1.16)		
Infection	3 (30)	4 (40)	3 (30)	10 (2.90)		
Others	19 (27.14)	43 (61.43)	8 (11.43)	70 (20.35)		
No complications	82 (32.28)	128 (50.39)	44 (17.32)	254 (73.84)		
Type of delivery						
Normal vaginal delivery	60 (34.09)	93 (82.84)	23 (13.07)	176 (51.16)	6.591	0.36
Episiotomy	22 (23.91)	51 (55.43)	19 (20.65)	92 (26.74)		
Cesarean mode of delivery	25 (34.24)	35 (47.94)	13 (17.81)	73 (21.22)		
Instrumental delivery	0 (0)	2 (66.67)	1 (33.33)	3 (0.87)		
Received family support during delivery						
Yes	89 (32.13)	144 (51.98)	44 (15.88)	277 (80.52)	0.724	0.696
No	18 (26.86)	37 (55.22)	12 (17.91)	67 (19.48)		
If yes, the relationship with the attendee						
Mother	58 (35.36)	83 (50.61)	23 (14.02)	164 (47.67)	4.582	0.801
Husband	18 (26.08)	38 (55.07)	13 (18.84)	69 (20.06)		
Other relatives	12 (30)	20 (50)	8 (20)	40 (11.63)		
Mother-in-law	1 (25)	3 (75)	0 (0)	4 (1.16)		
No relatives	18 (26.86)	37 (55.22)	12 (17.91)	67 (19.48)		
Family support received after delivery						
Yes	79 (30.74)	138 (53.69)	40 (15.56)	257 (74.71)	0.588	0.745
No	28 (32.18)	43 (49.42)	16 (18.39)	87 (25.29)		
If yes, the relationship with the attendee						
Mother	57 (33.93)	87 (51.78)	24 (14.28)	168 (48.84)	9.285	0.319
Husband	6 (14.28)	28 (66.67)	8 (19.05)	42 (12.21)		
Mother-in-law	14 (37.84)	18 (48.65)	5 (13.51)	37 (10.75)		
Other relatives	2 (20)	5 (50)	3 (30)	10 (2.90)		
No relatives	28 (32.18)	43 (49.42)	16 (18.39)	87 (25.29)		

Table 5 shows that there was a significant association found between received family support during delivery and the level of total support during the first trimester (p < 0.05). No significant association was found between gestational weeks completed at the time of delivery, presence of associated maternal complications/diseases during pregnancy, presence of close relatives before delivery, relationship with the attendee, presence of maternal complications during delivery, specific maternal complication, type of delivery, relationship with the attendee, family support received after delivery, relationship with the attendee, and maternal outcome with total family support during the first trimester (p > 0.05).

**Table 5:** Association between maternal outcome and the level of total support during the first trimester (N = 344) P value was calculated by the chi-square method N: sample size; n: number of participants included; PPH: postpartum hemorrhage

Maternal outcome	Poor support, n (%)	Moderate support, n (%)	Good support, n (%)	Total, n (%)	χ^2^ value	P value
Total gestational weeks completed at the time of delivery						
Below 36 weeks of gestation	21 (23.59)	40 (44.94)	28 (31.46)	89 (25.87)	2.873	0.579
37-40 weeks of gestation	39 (16.95)	123 (53.48)	68 (29.56)	230 (66.86)		
Above 40 weeks of gestation	6 (24)	12 (48)	7 (28)	25 (7.27)		
Presence of associated maternal complications/diseases during pregnancy						
Yes	14 (12.28)	62 (54.38)	38 (33.33)	114 (33.14)	5.306	0.07
No	52 (22.61)	113 (49.13)	65 (28.26)	230 (66.86)		
If yes, specify maternal complications						
PPH	0 (0)	3 (50)	3 (50)	6 (1.74)	10.998	0.202
Echlamsia	3 (7.89)	25 (65.79)	10 (26.31)	38 (11.04)		
Fever	1 (50)	0 (0)	1 (50)	2 (0.58)		
Other complications	11 (16.17)	33 (48.53)	24 (35.29)	68 (19.76)		
No complications	51 (22.17)	114 (49.56)	65 (28.26)	230 (66.86)		
Presence of close relatives before delivery						
Yes	46 (18.62)	131 (53.03)	70 (28.34)	247 (71.80)	1.702	0.427
No	20 (20.62)	44 (45.36)	33 (34.02)	97 (28.19)		
If yes, the relationship with the attendee						
Mother	31 (22.14)	75 (53.57)	34 (24.28)	140 (40.69)	6.274	0.393
Husband	12 (15)	40 (50)	28 (35)	80 (23.25)		
Other relatives	3 (11.11)	16 (59.26)	8 (29.63)	27 (7.85)		
No relatives	20 (20.62)	44 (45.36)	33 (34.02)	97 (28.19)		
Presence of associated maternal complications during delivery						
Yes	14 (15.55)	49 (54.44)	27 (30)	90 (26.16)	1.143	0.565
No	52 (20.47)	126 (49.60)	76 (29.92)	254 (73.83)		
If yes, specify maternal complications during delivery						
PPH	1 (16.67)	2 (33.33)	3 (50)	6 (1.74)	5.991	0.648
Echlamsia	1 (25)	3 (75)	0 (0)	4 (1.16)		
Infection	0 (0)	6 (60)	4 (40)	10 (2.90)		
Others	12 (17.14)	38 (54.28)	20 (28.57)	70 (20.35)		
No complications	52 (20.47)	126 (49.60)	76 (29.92)	254 (73.83)		
Type of delivery						
Normal vaginal delivery	34 (19.32)	94 (53.41)	48 (27.27)	176 (51.16)	9.512	0.147
Episiotomy	15 (16.30)	40 (43.48)	37 (40.21)	92 (26.74)		
Cesarean mode of delivery	17 (23.28)	38 (52.05)	18 (24.65)	73 (21.22)		
Instrumental delivery (forceps, vacuum)	0 (0)	3 (100)	0 (0)	3 (0.87)		
Received family support during delivery						
Yes	54 (19.49)	148 (53.43)	75 (27.07)	277 (80.52)	5.801	0.055
No	12 (17.91)	27 (40.30)	28 (41.79)	67 (19.47)		
If yes, the relationship with the attendee						
Mother	35 (21.34)	88 (53.66)	41 (25)	164 (47.67)	13.36	0.1
Husband	10 (14.49)	34 (49.27)	25 (36.23)	69 (20.06)		
Other relatives	9 (22.5)	22 (55)	9 (22.5)	40 (11.62)		
Mother-in-law	0 (0)	4 (100)	0 (0)	4 (1.16)		
No relatives	12 (17.91)	27 (40.29)	28 (41.79)	67 (19.47)		
Family support received after delivery						
Yes	44 (17.12)	137 (53.30)	76 (29.57)	257 (74.71)	3.49	0.175
No	22 (25.28)	38 (43.68)	27 (31.03)	87 (25.29)		
If yes, the relationship with the attendee						
Mother	35 (20.83)	86 (51.19)	47 (27.97)	168 (48.83)	11.992	0.152
Husband	4 (9.53)	21 (50)	17 (40.47)	42 (12.20)		
Mother-in-law	5 (13.51)	24 (64.86)	8 (21.62)	37 (10.75)		
Other relatives	0 (0)	6 (60)	4 (40)	10 (2.90)		
No relatives	22 (25.28)	38 (43.68)	27 (31.03)	87 (25.29)		

Table 6 indicates that there was no significant association between the health of the newborn at birth, birth weight, height, abnormalities, complications, sex of the baby, received family support for feeding, relationship with the attendee, and physical support with the fetal outcome during the first trimester (p > 0.05).

**Table 6:** Association between fetal outcome and the level of physical support during the first trimester (N = 344) P value was calculated by the chi-square method N: sample size; n: number of participants included; LBW: low birth weight

Fetal outcome	Poor support, n (%)	Moderate support, n (%)	Good support, n (%)	Total, n (%)	χ^2^ value	P value
Healthy newborn has born						
Yes	78 (30.11)	141 (54.44)	40 (15.44)	259 (75.29)	1.441	0.486
No	29 (34.11)	40 (47.06)	16 (18.82)	85 (24.71)		
Birth weight of the baby at delivery						
Below 1.5 kg	0 (0)	2 (66.67)	1 (33.33)	3 (0.87)	8.519	0.203
1.5-2 kg	30 (27.02)	55 (49.55)	26 (23.42)	111 (32.26)		
2-2.5 kg	10 (31.25)	17 (53.12)	5 (15.62)	32 (9.30)		
Above 2.5 kg	67 (33.84)	107 (54.04)	24 (12.12)	198 (57.56)		
Length of the present baby						
≤48 cm	20 (30.77)	35 (53.84)	10 (15.38)	65 (18.89)	0.881	0.927
49-50 cm	29 (32.22)	44 (48.89)	17 (18.89)	90 (26.16)		
Above 50 cm	58 (30.68)	102 (53.97)	29 (15.34)	189 (54.94)		
Any abnormality in the present baby						
Yes	55 (31.79)	95 (54.91)	23 (13.29)	173 (50.29)	2.306	0.316
No	52 (30.40)	86 (50.29)	33 (19.30)	171 (49.70)		
Specify complications						
LBW	12 (34.28)	13 (37.14)	10 (28.57)	35 (10.17)	9.402	0.31
Fetal distress	6 (26.08)	11 (47.82)	6 (26.08)	23 (6.68)		
Birth asphyxia	2 (20)	6 (60)	2 (20)	10 (2.90)		
Other complications	7 (24.13)	18 (62.07)	4 (13.79)	29 (8.43)		
No complications	80 (32.39)	133 (53.84)	34 (13.76)	247 (71.80)		
Sex of the present baby						
Male	55 (31.79)	95 (54.91)	23 (13.29)	173 (50.29)	2.306	0.316
Female	52 (30.41)	86 (50.29)	33 (19.30)	171 (49.71)		
Received family support for baby care during feeding						
Yes	72 (31.17)	122 (52.81)	37 (16.01)	231 (67.15)	4.976	0.083
No	26 (23.01)	74 (65.48)	13 (11.50)	113 (32.84)		
Relationship with the attendee supporting baby care						
Mother	30 (30)	50 (50)	20 (20)	100 (29.07)	13.826	0.086
Husband	19 (30.16)	34 (53.97)	10 (15.87)	63 (18.31)		
Mother-in-law	16 (41.02)	17 (43.59)	6 (15.38)	39 (11.33)		
Others	7 (24.13)	21 (72.41)	1 (3.45)	29 (8.43)		
No relatives	26 (23.01)	74 (65.48)	13 (11.50)	113 (32.85)		

From Table 7, it was found that there was no significant association between the following factors and fetal outcome during the first trimester: birth status, birth weight, height, any abnormality in the baby, any complications, sex of the present baby, received family support for baby care, relationship with the attendee, and emotional support (p > 0.05).

**Table 7:** Association between fetal outcome and the level of emotional support during the first trimester (N = 344) P value was calculated by the chi-square method N: sample size; n: number of participants included; LBW = low birth weight; FD: full-term delivery

Fetal outcome	Poor support, n (%)	Moderate support, n (%)	Good support, n (%)	Total, n (%)	χ^2^ value	P value
Healthy newborn has born						
Yes	82 (31.66)	127 (49.03)	50 (19.30)	259 (75.29)	0.128	0.938
No	28 (32.94)	42 (49.41)	15 (17.64)	85 (24.71)		
Birth weight of the baby at delivery						
Below 1.5 kg	0 (0)	3 (100)	0 (0)	3 (0.87)	5.545	0.476
1.5-2 kg	31 (27.92)	56 (50.45)	24 (21.62)	111 (32.26)		
2-2.5 kg	11 (34.37)	17 (53.12)	4 (12.5)	32 (9.30)		
Above 2.5 kg	68 (69.38)	93 (94.89)	37 (37.75)	98 (28.49)		
Length of the present baby						
Below or equal to 48 cm	22 (33.84)	31 (47.69)	12 (18.46)	65 (18.89)	0.652	0.957
49-50 cm	30 (33.33)	45 (50)	15 (16.67)	90 (26.16)		
Above 50 cm	58 (30.68)	93 (49.20)	38 (20.10)	189 (54.94)		
Any abnormality in the present baby						
Yes	30 (30.92)	52 (53.60)	15 (15.46)	97 (28.19)	1.44	0.487
No	80 (32.39)	117 (47.37)	50 (20.24)	247 (71.80)		
Specify complications						
LBW	13 (37.14)	17 (48.57)	5 (14.28)	35 (10.17)	4.277	0.831
FD	7 (30.43)	13 (56.52)	3 (13.04)	23 (6.68)		
Birth asphyxia	1 (10)	7 (70)	2 (20)	10 (2.90)		
Other complications	9 (31.03)	15 (51.72)	5 (17.24)	29 (8.43)		
No complications	80 (32.39)	117 (47.37)	50 (20.24)	247 (71.80)		
Sex of the present baby						
Male	55 (31.79)	84 (48.55)	34 (19.65)	173 (50.29)	0.133	0.936
Female	55 (32.16)	85 (49.71)	31 (18.13)	171 (49.71)		
Received family support for baby care during feeding						
Yes	71 (30.74)	120 (51.95)	40 (17.32)	231 (67.15)	2.405	0.3
No	39 (34.51)	49 (43.36)	25 (22.12)	113 (32.85)		
Relationship with the attendee supporting baby care						
Mother	32 (32)	52 (52)	16 (16)	100 (29.07)	6.632	0.577
Husband	16 (25.40)	34 (53.97)	13 (20.63)	63 (18.31)		
Mother-in-law	10 (25.64)	23 (58.97)	6 (15.38)	39 (11.34)		
Others	12 (41.38)	12 (41.38)	5 (17.24)	29 (8.43)		
No relatives	40 (35.40)	48 (42.48)	25 (22.12)	113 (32.85)		

From Table 8, it was found that there was a significant association between the level of psychosocial support and the attendee supporting baby care (p < 0.05). However, no significant association was found between psychosocial support during the first trimester and the following factors: health of the newborn at birth; weight of baby at delivery; length of the present baby; any abnormalities in the baby; if yes, specify abnormality; the baby's sex; and fetal outcome (p > 0.05).

**Table 8:** Association between fetal outcome and the level of psychosocial support during the first trimester (N = 344) P value was calculated by the chi-square method N: sample size; n: number of participants included; LBW: low birth weight; FD: full-term delivery

Fetal outcome	Poor support, n (%)	Moderate support, n (%)	Good support, n (%)	Total, n (%)	χ^2^ value	P value
Healthy newborn has born						
Yes	78 (30.12)	141 (54.44)	40 (15.44)	259 (75.29)	1.441	0.486
No	29 (34.12)	40 (47.06)	16 (18.82)	85 (24.71)		
Birth weight of the baby at delivery						
Below 1.5 kg	0 (0)	2 (66.67)	1 (33.33)	3 (0.87)	8.519	0.203
1.5-2 kg	30 (27.03)	55 (49.55)	26 (23.42)	111 (32.27)		
2-2.5 kg	10 (31.25)	17 (53.13)	5 (15.63)	32 (9.30)		
Above 2.5 kg	67 (33.84)	107 (54.04)	24 (12.12)	198 (57.56)		
Length of the present baby						
Below or equal to 48 cm	20 (30.77)	35 (53.85)	10 (15.38)	65 (18.90)	0.881	0.927
49-50 cm	29 (32.22)	44 (48.89)	17 (18.89)	90 (26.16)		
Above 50 cm	58 (30.69)	102 (53.97)	29 (15.34)	189 (54.94)		
Any abnormality in the present baby						
Yes	27 (27.84)	48 (49.48)	22 (22.68)	97 (28.20)	4.117	0.128
No	80 (32.39)	133 (53.85)	34 (13.77)	247 (71.80)		
Specify complications						
LBW	12 (34.29)	13 (37.14)	10 (28.57)	35 (10.17)	9.402	0.31
FD	6 (26.09)	11 (47.83)	6 (26.09)	23 (6.69)		
Birth asphyxia	2 (20)	6 (60)	2 (20)	10 (2.91)		
Other complications	7 (24.14)	18 (62.07)	4 (13.79)	29 (8.43)		
No complications	80 (32.39)	133 (53.85)	34 (13.77)	247 (71.80)		
Sex of the present baby						
Male	55 (31.79)	95 (54.91)	23 (13.29)	173 (50.29)	2.306	0.316
Female	52 (30.41)	86 (50.29)	33 (19.30)	171 (49.71)		
Received family support for baby care during feeding						
Yes	66 (28.57)	124 (53.68)	41 (17.75)	231 (67.15)	2.535	0.282
No	41 (36.28)	57 (50.44)	15 (13.27)	113 (32.85)		
Relationship with the attendee supporting baby care						
Mother	31 (31)	53 (53)	16 (16)	100 (29.07)	16.052	0.042
Husband	12 (19.05)	35 (55.56)	16 (25.40)	63 (18.31)		
Mother-in-law	18 (46.15)	15 (38.46)	6 (15.38)	39 (11.34)		
Others	5 (17.24)	20 (68.97)	4 (13.79)	29 (8.43)		
No relatives	41 (36.28)	58 (51.33)	14 (12.39)	113 (32.85)		

Table 9 shows that no significant association was found between the length of the present baby, its sex, the relationship with the attendee supporting baby care, and the level of total family support during the first trimester (p > 0.05).

**Table 9:** Association between fetal outcome and the level of total family support during the first trimester (N = 344) P value was calculated by the chi-square method N: sample size; n: number of participants included; LBW: low birth weight; FD: full-term delivery

Fetal outcome	Poor support, n (%)	Moderate support, n (%)	Good support, n (%)	Total, n (%)	χ^2^ value	P value
Healthy newborn has born						
Yes	49 (18.92)	130 (50.19)	80 (30.89)	259 (75.29)	0.447	0.8
No	17 (20)	45 (52.94)	23 (27.06)	85 (24.71)		
Birth weight of the baby at delivery						
Below 1.5 kg	0 (0)	1 (33.33)	2 (66.67)	3 (0.87)	6.412	0.379
1.5-2 kg	19 (17.12)	54 (48.65)	38 (34.23)	111 (32.27)		
2-2.5 kg	8 (25)	19 (59.38)	5 (15.63)	32 (9.30)		
Above 2.5 kg	39 (19.70)	101 (51.01)	58 (29.29)	198 (57.56)		
Length of the present baby						
Below or equal to 48 cm	11 (16.92)	36 (55.38)	18 (27.69)	65 (18.90)	1.147	0.887
49-50 cm	18 (20)	47 (52.22)	25 (27.78)	90 (26.16)		
Above 50 cm	37 (19.58)	92 (48.68)	60 (31.75)	189 (54.94)		
Any abnormality to the present baby						
Yes	17 (17.53)	48 (49.48)	32 (32.99)	97 (28.20)	0.664	0.717
No	49 (19.84)	127 (51.42)	71 (28.74)	247 (71.80)		
Specify complications						
LBW	7 (20)	18 (51.43)	10 (28.57)	35 (10.17)	2.429	0.965
FD	5 (21.74)	10 (43.48)	8 (34.78)	23 (6.69)		
Birth asphyxia	1 (10)	6 (60)	3 (30)	10 (2.91)		
Other complications	4 (13.79)	14 (48.28)	11 (37.93)	29 (8.43)		
No complications	49 (19.84)	127 (51.42)	71 (28.74)	247 (71.80)		
Sex of the present baby						
Male	33 (19.08)	92 (53.18)	48 (27.75)	173 (50.29)	0.927	0.629
Female	33 (19.30)	83 (48.54)	55 (32.16)	171 (49.71)		
Received family support for baby care during feeding						
Yes	47 (20.35)	112 (48.48)	72 (31.17)	231 (67.15)	1.635	0.442
No	19 (16.81)	63 (55.75)	31 (27.43)	113 (32.85)		
Relationship with the attendee supporting baby care						
Mother	21 (21)	43 (43)	36 (36)	100 (29.07)	11.073	0.198
Husband	12 (19.05)	28 (44.44)	23 (36.51)	63 (18.31)		
Mother-in-law	10 (25.64)	21 (53.85)	8 (20.51)	39 (11.34)		
Others	4 (13.79)	20 (68.97)	5 (17.24)	29 (8.43)		
No relatives	19 (16.81)	63 (55.75)	31 (27.43)	113 (32.85)		

## Discussion

In this research study, 56 participants (16.3%) received good psychosocial support, 181 (52.6%) received moderate support, and 107 (31.1%) received poor psychosocial support. The present study demonstrated results similar to those of a study conducted by Abdollahpour et al. [7]. The study used the perceived social support from the family scale and found that 1.3% of women had poor family support, 27.9% had moderate family support, and 69% had good family support. In both studies, the majority of women received moderate support. Due to family support, early diagnosis and prevention of any complications can be ruled out.

In this study, no significant association was found between the types of delivery, complications during delivery, received family support during delivery, relationship with the attendee, and family support received after delivery with psychosocial support in the first trimester of pregnancy (p > 0.05). These findings are similar to those of Allendorf [8], who investigated the quality of family relationships and maternal health. It was found that there was no significant correlation between social support and the type of delivery, birth weight, number of prenatal care visits, and obstetric complications (p > 0.05). These results suggest that while social support, including family support, may not significantly impact specific delivery outcomes, it is still crucial for overall maternal well-being during pregnancy.

This study's findings show that during the first trimester, women need support due to morning sickness and other physiological changes. The results of the study by Lutterodt et al. [9] indicated that in the first trimester, most women experienced more than one symptom. While many women accepted these symptoms, those involving pain or bleeding were particularly concerning, and nausea frequently caused minor worries for about one-fifth of women. During pregnancy, women need assistance and support from their families and care providers to address their worries. Our study found a significant association between pregnancy acceptance and physical support during the first trimester (p < 0.05).

In the present study, good family support had shown an impact on maternal and fetal outcomes, similar to those who received support and had normal delivery. The study showed that out of 344 participants, only 80 (23.26%) husbands supported pregnant women during the first trimester, providing emotional security, mental peace, and improved physical health. A study by Sokoya et al. in Nigeria demonstrated that 86% of the women who were supported by their husbands experienced less stress during pregnancy, feeling emotionally secure and physically healthy [10]. A recent study found that the importance of family support during the first trimester of pregnancy can enhance maternal and fetal outcomes. This period is marked by significant physical and emotional changes, making it a vulnerable time for expectant mothers. In the present study, good family support was shown to impact maternal and fetal outcomes positively.

This research found a significant association between psychosocial support received in the first trimester and total gestational weeks completed at the time of delivery (p < 0.05). This study selected both multi and primigravida mothers. A majority of 195 respondents (56.6%) were identified as housewives and indicated that they rely on family members to make decisions regarding their visits to the doctor. A study conducted by Prabhu et al. [11] selected both multi and primigravida mothers; there was a substantial association between maternal age and prenatal depression; the majority of the study participants were housewives who were financially dependent on their partners.

In the current study, it is important to note that support for women from both their husbands and their in-laws is crucial. It will help to achieve healthy maternal and fetal outcomes. Out of 344 participants, 168 (48.83%) received support from mothers, 42 (12.20%) from husbands, 37 (10.75%) from mother-in-laws, and 10 women (2.91%) received support from other relatives. In this study, it is demonstrated that 87 women (25.29%) did not receive support from any of their relatives. Fatigue experienced during pregnancy encompasses physical, psychological, and emotional aspects, as indicated by Naz et al. [12]. Healthy pregnancy outcomes are directly proportional to the care taken by mother-in-laws and husbands, who are the key people. They felt helpless when no one listened to their health problems [12-14].

Policy implications for family support during pregnancy: Research suggests that promoting family involvement and support during pregnancy is crucial for optimal maternal and child health outcomes. Healthcare policies and interventions should be evidence-based and designed to foster an environment where families are actively engaged and supported throughout the pregnancy journey. By adopting these recommendations, healthcare systems can improve health outcomes for both mothers and their babies, ultimately contributing to healthier families and communities.

## Conclusions

The study's conclusion highlights the significant role of family support in achieving positive maternal and fetal outcomes during pregnancy. Specifically, when women experience symptoms like morning sickness and physiological changes during the first trimester, family support becomes crucial for ensuring good outcomes. The study suggests that receiving adequate family support during this period is associated with improved maternal and fetal health, as evidenced by a higher likelihood of normal delivery among women who received support. As reported by study participants, the impact of the husband's support was perceived to provide emotional security, mental peace, and improved physical health for pregnant women. However, the study also reveals that only a minority of husbands (23.26%) provided support during the first trimester, indicating a potential area for improvement in terms of spousal support during pregnancy. Furthermore, the study identifies a significant association between attendees supporting baby care and the level of psychosocial support (p < 0.05). This underscores the importance of psychosocial support during the first trimester, highlighting the need for interventions that address the psychological and social well-being of pregnant women. The study emphasizes the vital role of family support, particularly from husbands, in ensuring positive maternal and fetal outcomes during pregnancy, especially during the first trimester.
